# Wellness and Prevalence of Hypogonadism Among Male Resident Physicians

**DOI:** 10.5152/tud.2025.24121

**Published:** 2025-04-04

**Authors:** Basil F. Mirza, Gene Austin Krishingner, Kevin J. Campbell

**Affiliations:** 1 University of Florida College of Medicine, Florida, USA; 2 Department of Urology, University of Florida College of Medicine, Florida, USA

**Keywords:** Hypogonadism, men’s health, health promotion, resident physician

## Abstract

**Objective:**

Hypogonadism is estimated to affect up to 30% of men aged 40-70, with a range of symptoms and health implications. Despite its prevalence, its impact on resident physicians, a group known to experience high levels of stress, irregular sleep patterns, and long working hours, remains largely unexplored. This study aimed to evaluate the prevalence and impact of hypogonadism among male resident physicians.

**Methods:**

A prospective study was conducted involving male physicians training at a tertiary referral institution. Participation was offered through distribution to Accreditation Council for Graduate Medical Education (ACGME) program coordinators and program directors. Participants were given the Androgen Deficiency in the Aging Male (ADAM) and 36-Item Short Form Health Survey questionnaires, and underwent labs including testosterone, follicle-stimulating hormone (FSH), luteinizing hormone, and estradiol levels.

**Results:**

Of the 651 male trainees, 27 residents were interested in participating for a response rate of 4.1%. Sixty-seven percent (18/27) completed the surveys and 22% (6/27) completed lab work. Two-thirds of the participants who completed the lab work had total testosterone levels below 400 ng/dL, and half of the participants scored positively on the ADAM questionnaire. A total of 44.4% of participants reported that their health was worse compared to a year prior.

**Conclusion:**

Male resident physicians are an at-risk population for symptoms and signs of hypogonadism. This population may benefit from screening and interventions aimed at reducing the impact of hypogonadism. The findings of this study underscore the need for further research to confirm these results and explore potential interventions.

**Main Points**

- Prevalence of hypogonadism signs and symptoms may be higher among male resident physicians compared to the general population.
- There is significant overlap in the symptoms of hypogonadism and resident burnout, making the diagnosis more challenging.
- Male resident physicians and training programs would likely benefit from awareness of the prevalence of hypogonadism and should encourage awareness and screening when necessary.

## Introduction

Hypogonadism is a significant medical condition that has gained increased attention in recent years. The prevalence of hypogonadism varies widely, with estimates from 2.1% to 30% in men aged 40-79 years, depending on the definition and population studied.^1^ The severity of hypogonadism can be classified as mild to severe, with symptoms including reduced libido, erectile dysfunction, decreased energy, depression, and anemia, among others.^2^ The economic burden of hypogonadism is substantial, with 1 study estimating the 20-year cost of testosterone deficiency in US men aged 45 to 74 at $190-$525 billion.^3^ The importance of addressing hypogonadism is underscored by its potential long-term health impacts, including an increased risk of cardiovascular disease and osteoporosis.^4^ Understanding and addressing hypogonadism is crucial for improving patient quality of life and reducing healthcare costs.

Despite the growing recognition of hypogonadism as a significant health issue, its prevalence and impact in young adult males remain understudied. Resident physicians, the majority of whom are under the age of 45, constitute a group known to experience high levels of stress, irregular sleep patterns, and long working hours. These factors are associated with hypogonadism in other populations, but their impact on resident physicians remains largely unexplored. Non-standard shift work and insufficient sleep have previously been linked to urologic complications in males.^5^,^6^ Furthermore, the symptoms of hypogonadism, such as fatigue, depression, and decreased libido, overlap significantly with symptoms of burnout and depression, conditions known to be prevalent among resident physicians.^7^ This overlap may lead to misdiagnosis or underdiagnosis of hypogonadism in this population, potentially exacerbating the health and wellness challenges they face. Clinical diagnosis for adults under age 45 can be further complicated, as they often present with non-specific symptoms, such as fatigue rather than overt sexual dysfunction. Recent evidence also shows that the cutoff of 300 ng/dL used to determine testosterone deficiency at any age may be inappropriately low to accurately diagnose hypogonadism in young adults, with a cutoff of 400 ng/dL likely being more appropriate for men aged 20 to 44.^8^ Therefore, the objective of this pilot study was to evaluate the prevalence and impact of hypogonadism among male resident physicians, with the aim of improving health outcomes and wellness in this at-risk population.

The primary objective of the study was to evaluate the prevalence of hypogonadism among male resident physicians. Given the stressful and demanding nature of their work, it is hypothesized that this population may be at a higher risk for developing hypogonadism. By assessing the prevalence of this condition, the aim is to shed light on an underexplored aspect of resident physician health and wellness. Secondary objectives of the study included assessing the severity of hypogonadism symptoms in this population and exploring the potential relationship between hypogonadism and other health issues common among resident physicians, such as burnout and depression. By achieving these objectives, the hope is to provide a more comprehensive understanding of the health challenges faced by resident physicians and inform strategies for improving their health and wellness.

## Material and Methods

### Data Source

We conducted a prospective study involving male physicians in training at the institution. The study was initiated following approval by the University of Florida Institutional Review Board (IRB 202201215, 15 December 2022). The data for the study were obtained through a combination of survey questionnaires and laboratory tests. The survey data were collected using the Research Electronic Data Capture (RedCAP) system.

### Study Subjects

Participants were identified through study protocol distribution to ACGME resident and fellow program physicians. The study population consisted of 651 male residents and fellows. Of these, 27 residents expressed interest in participation, yielding a response rate of 4.1%. Prior to participation, informed consent was obtained from each subject. The participants were not restricted by age, specialty, and post-graduation year (PGY). Incentives to participate included receiving lab results and follow-up through the institution, in congruence with the remainder of their healthcare and any necessary workup of abnormal results. Enrolled subjects received reminders and follow-up notifications via email and their electronic health record.

### Exposures

The exposure variables of interest in this study were the working conditions of the resident physicians. These variables were assessed through the participants’ specialty and PGY level, as these factors are known to influence the working conditions of resident physicians. Call and night shift work within the preceding months were also measured to quantify atypical sleep schedules. Other lifestyle factors and medical history were not assessed in the survey at this time.

### Outcomes

The primary outcome of interest was the prevalence of hypogonadism among the participants, as measured by testosterone levels in the blood. Participants were asked to complete lab work, including a basic metabolic panel, complete blood count, lipid panel, thyroid panel, and measurements of testosterone, follicle-stimulating hormone, luteinizing hormone, and estradiol. Secondary outcomes included the severity of hypogonadism symptoms, as measured by the Androgen Deficiency in the Aging Male (ADAM) questionnaire, and overall health status, as measured by the 36-Item Short Form Health Survey (SF-36).

### Statistical Analysis

We used descriptive statistics to summarize the baseline demographics and laboratory results of the participants. The prevalence of hypogonadism was calculated as the proportion of participants with total testosterone levels below 400 ng/dL.

## Results

The characteristics of the study subjects are presented in Table 1. Out of the 651 male house staff contacted, 27 responded, yielding a response rate of 4.1%. Of these, 88.9% (24/27) provided demographic data, and 66.7% (18/27) completed the surveys. Among those who filled out the survey, 33.3% (6/18) completed the lab work. The participants were diverse in terms of age, specialty, and PGY. These values were reported independently in Table 1 to maintain anonymity.

The results of the laboratory tests are presented in Table 2. Two-thirds (4/6) of the participants who completed the lab work had total testosterone levels below 400 ng/dl. Two participants met the serum criteria for hypogonadism.

The results of the ADAM and SF-36 questionnaires are presented in Table 3. Half (9/18) of the participants scored positively on the ADAM questionnaire, indicating the presence of hypogonadal symptoms. Furthermore, 44.4% (8/18) of the participants reported that their health was worse compared to a year prior.

Among the participants that reported not taking any overnight call, 20% (1/5) scored positively on the ADAM questionnaire. For those that do take any form of overnight call, 61.5% (8/13) scored positively.

## Discussion

The key finding of this study was the presence of hypogonadism among male resident physicians. Two-thirds of the participants who completed the lab work had total testosterone levels below 400 ng/dL, and half of the participants scored positively on the ADAM questionnaire, suggesting a high prevalence of hypogonadism symptoms. Additionally, residents who reported taking overnight calls were more likely to score positively.

These findings are consistent with previous research showing a link between stressful working conditions and hypogonadism. However, they extend the existing literature by demonstrating this link in a population of resident physicians, a group that has not been extensively studied in this context. The findings also highlight the potential overlap between hypogonadism and other health issues common among resident physicians, such as burnout and depression.

The study has several strengths, including the use of both questionnaires and laboratory tests to assess the prevalence and impact of hypogonadism. However, there are limitations that should be considered, and the results should be viewed as pilot data that can inform future research. Because of the nature of the study as a survey of trainees at the institution, a control group (i.e., age-matched non-physician males) was not included, which limits the conclusions that can be drawn from the data. The sample size and response rate were relatively small, and the participants were not randomly selected, which limits the generalizability of the findings. With a 22% rate of completion of lab work, the study likely cannot accurately provide an estimate for the prevalence of hypogonadism in resident physicians. Furthermore, potential confounding factors such as age, lifestyle factors, and underlying health conditions were not controlled for. These variables, especially underlying conditions such as obesity and diabetes, have known associations with hypogonadism. In an expanded study with longitudinal follow-up, participants would be stratified based on predisposing factors. However, for the purposes of this small pilot study, these limitations are unlikely to have affected the main conclusion regarding the presence of hypogonadism among resident physicians. The population remained heterogeneous within the resident cohort, as there was not enough participation to stratify by study discipline. Given a larger cohort, stratifying by specialty or PGY level may reveal important distinctions in hypogonadal symptoms among groups. Additionally, only 1 time point was recorded per trainee, which limits the exploration of progression or resolution of symptoms. A secondary call for follow-up at 6 months was requested; however, the university had instituted a separate health screening event.

The findings of this study have important implications for the health and wellness of resident physicians. The pilot data suggest that routine screening for hypogonadism may be beneficial in this population. Future research should aim to confirm the findings in larger, more diverse samples of resident physicians and to explore potential interventions to reduce the prevalence and impact of hypogonadism. These future studies should also include longitudinal follow-up that assesses clinical changes in individual subjects over time compared to changes in lifestyle or reported stressors.

It was found that hypogonadism is prevalent among male resident physicians. Given the findings, the use of the ADAM, SF-36, or similar surveys by residency programs may prompt appropriate workup and treatment for a significant number of trainees. Many programs now provide screening and interventions for conditions like depression and burnout in residents, but it is important to recognize hypogonadism as a potential cause of symptoms and to encourage proper screening with tools specific to this issue. This at-risk population would likely benefit from wellness screening and interventions aimed at reducing the prevalence and impact of hypogonadism.

## Figures and Tables

**Table 1.** Participant Characteristics (n = 24)

	n	%
Specialty		
Critical Care	3	12.5
Dermatology	4	16.7
Radiology	1	4.2
Orthopedics	1	4.2
Neurology	1	4.2
Anesthesia	9	37.5
Emergency	1	4.2
Surgery	2	8.3
Urology	1	4.2
Oral Surgery	1	4.2
PGY		
1	5	20.8
2	3	12.5
3	5	20.8
4	5	20.8
5	2	8.3
6	3	12.5
9	1	4.2

**Table 2.** Results of Serum Testosterone Testing

Subject	Serum Testosterone (ng/dL)	Positive ADAM? (Y/N)
1	398	N
2	612	Y
3	298	Y
4	305	Y
5	339	N
6	849	N

**Table 3.** Androgen Deficiency in the Aging Male (ADAM) Positivity Rates and Short Form 36 (SF-36) Questionnaire Averages For All Study Participants and Those Who Obtained Lab Work with Hypogonadal vs. Eugonadal Results. For the SF-36, 100% represents the highest level of function

	Hypogonadal (n = 2) (%)	Eugonadal (n = 4) (%)	All Participants (n = 18) (%)
Positive ADAM	100	25	50
SF-36			
Physical Functioning	82.5	95.0	94.4
Role Limitations Due To Physical Health	62.5	75.0	73.6
Role Limitations Due To Emotional Problems	33.3	75.0	59.3
Energy/Fatigue	32.5	51.3	43.1
Emotional Well-Being	60.0	66.0	64.7
Social Functioning	62.5	68.8	70.1
Pain	68.8	87.5	81.0
General Health	35.0	61.3	59.4
Health Change	37.5	43.8	42.6

## Data Availability

The data that support the findings of this study are available from the corresponding author upon reasonable request.
